# Influence of bovine serum albumin on corrosion behaviour of pure Zn in phosphate buffered saline

**DOI:** 10.1007/s10856-021-06567-x

**Published:** 2021-08-18

**Authors:** Lijun Liu, Lili Lu, Hai-Jun Zhang, Lu-Ning Wang

**Affiliations:** 1 Beijing Advanced Innovation Centre for Materials Genome Engineering, School of Materials Science and Engineering, University of Science and Technology Beijing, Beijing, 100083 China; 2 National United Engineering Laboratory for Biomedical Material Modification, Qihe, Shandong 251100 China; 3 State Key Laboratory of Advanced Metals and Materials, University of Science and Technology Beijing, Beijing, 100083 China

## Abstract

Zinc (Zn) and its alloys have received increasing attention as new alternative biodegradable metals. However, consensus has not been reached on the corrosion behaviour of Zn. As cardiovascular artery stent material, Zn is supposed to contact with plasma that contains inorganic salts and organic components. Protein is one of the most important constitute in the plasma and could adsorb on the material surface. In this paper, bovine serum albumin (BSA) was used as a typical protein. Influences of BSA on pure Zn corrosion in phosphate buffered saline is investigated as a function of BSA concentrations and immersion durations by electrochemical techniques and surface analysis. Results showed that pure Zn corrosion was progressively accelerated with BSA concentrations (ranging from 0.05 to 5 g L^−1^) at 0.5 h. With time evolves, formation of phosphates as corrosion product was delayed by BSA adsorption, especially at concentration of 2 g L^−1^. Within 48 h, the corrosion of pure Zn was alleviated by BSA at concentration of 0.1 g L^−1^, whereas the corrosion was enhanced after 168 h. Addition of 2 g L^−1^ BSA has opposite influence on the pure Zn corrosion. Furthermore, schematic corrosion behaviour at protein/Zn interfaces was proposed. This work encourages us to think more about the influence of protein on the material corrosion and helps us to better understand the corrosion behaviour of pure Zn.

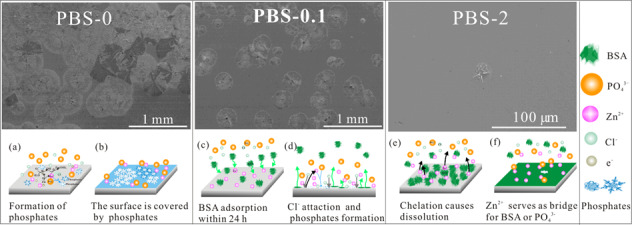

## Introduction

Zinc (Zn) and its alloys have received increasing attention as new alternative biodegradable metals, due to their biological merits [1–8] and ideal degradation behaviour [9–13]. However, biocompatibility and mechanical integrity of implantations are often correlated to corrosion behaviour including corrosion rate and corrosion products [14].

Compared with biodegradable iron (Fe) and magnesium (Mg), Zn-based metals have moderate corrosion rates [15]. By using Zn alloys, many of severe issues associated with Mg and Fe could be avoided, such as hydrogen accumulation and localized corrosion [1, 16]. Up to date, numerous efforts were devoted to explore the corrosion mechanism of Zn alloys as biomaterials [17–19]. However, consensus has not been reached since the corrosion behaviour of Zn is affected by various factors, such as materials (alloying elements, surface conditions, etc), surrounding environments (chemical compositions, oxygen concentrations and pH) as well as the construction (implant design). And in vitro results have often been shown to be influenced by the choice of test solution [20]. Pure Zn displayed uniform corrosion with corrosion products of ZnO, Zn(OH)_2_, and Zn_5_(OH)_8_Cl_2_·H_2_O when exposed to saline [17]. In phosphate buffer saline (PBS) and Hank’s solution, pure Zn was uniformly corroded. And in a long-term assay, localized corrosion was observed and corrosion rate was accelerated [2, 18]. Pure Zn surface was covered by a passivation film containing biomolecules and inorganic components immersed in both whole blood and plasma. And the corrosion rate differs from that in PBS and Ringer’s solution, which only contain inorganic components [21].

Implants are in contact with tissue medium and blood in the body, which contain salts as well as organic molecules [22–24]. So far role of organic compounds is not clearly addressed in the regulation of Zn-based alloys degradation. Protein is known as a main component of organic molecules and is the first to interact with implants [25], altering corrosion behaviour of metals by either adsorption or chelation [26, 27] and then affecting the biocompatibility of materials [28]. The major protein in serum is albumin that is present typically around 50 g L^−1^, taking up about 60% of the total protein [29]. Previous study reported that bovine serum albumin (BSA) has been used to simulated the protein in human body due to the strong similarity of structure to human serum albumin [30, 31]. For example, in saline, BSA acting as a cathodic inhibitor reduced the corrosion rate of both Mg1.5Ca alloy and pure Mg [32, 33]. In simulated body fluid (SBF), 40 g L^−1^ BSA can slow down the corrosion of Mg-Mn alloy in the initial stage. By extending immersion time, BSA changed the corrosion mode from localized corrosion to general corrosion and increased the corrosion rate [34]. Similar result on Mg-1.5Zn-0.6Zr-0.2Sc alloy was obtained in Hank’s solution [35]. The change of BSA concentration also affected the corrosion of materials. For instance, BSA with concentration of 40 g L^−1^ enhanced the corrosion of AZ80 alloy due to the self-aggregation or chelation with metals. However, addition of 10 and 20 g L^−1^ BSA doses could alleviate the corrosion because of the adsorption on alloy surfaces [22]. Beneficial or detrimental effects of BSA on the corrosion have been found on inert biomaterials as well, which are related to immersion time, BSA concentration, ionic strength, temperature, etc [14, 36, 37]. The hypothesis of this study is that variation of protein concentration and duration of immersion could also affect the corrosion behaviour of pure Zn as blood vessel stent materials.

Although Zn is considered as a promising biodegradable material, there is relatively limited source of literature found on the electrochemical behaviour of pure Zn in albumin containing solutions. In our previous work [38], the corrosion behaviour of pure Zn has been evaluated in artificial plasma up to 28 days by immersion test and electrochemical measurements. In the presence of BSA (60 g L^−1^), the corrosion rate of pure Zn was distinctly reduced and the corrosion product was consisted of BSA-ZnO/Zn(OH)_2_ complex rather than phosphates that found in revised simulated body fluid [19]. Thus, it is likely that the presence of albumin significantly affects the interaction at zinc/electrolyte interface and further the degradation behaviour of zinc in certain culture, which has not been elucidated in detail. Herein, electrochemical behaviour of pure Zn and adsorption of BSA was investigated in PBS with several BSA concentrations. Surface morphology and corrosion products composition of pure Zn were analysed by SEM, EDS, and XPS. The study will provide an important basis for deeply understanding the interfacial behaviour of proteins and Zn-based biomaterials in vivo.

## Materials and methods

### Sample preparation

Pure Zn sheets (2 mm in thickness) with 99.99% purity were purchased from China New Metal Materials Technology Co. Ltd. The samples for immersion test were cut into disks (10 mm in diameter) and ground by SiC sandpapers, then mechanically polished with diamond abrasive paste to get a mirror surface. Surface contaminants were removed by successively rinse in deionized water, acetone and ethanol. Afterwards, the samples were dried by a dryer and then soaked in testing solutions.

### Immersion test

The immersion tests were carried out in PBS with BSA (98%, Beijing Nokasn, Fig. S1) ranged 0 g L^−1^ to 5 g L^−1^. The composition of each solution is shown in Table S1. All the solutions were prepared with deionized water. The pH was adjusted by 1 M NaOH or HCl to 7.4 ± 0.05 before immersion. Each disk was separately soaked in 40 mL solution in a centrifuge tube with sealed cap and then put into an incubator to keep the temperature at 37 ± 0.5 ^o^C. The solution was renewed every 24 h. At different immersion intervals (0.5, 5, 24, 48, 168 and 336 h), the samples were picked up and washed with distilled water, then dried for the subsequent characterization.

### Surface and component analysis

Surface morphologies of pure Zn were characterized by a field emission scanning electron microscope (FE-SEM, FEI Quanta 200), coupled with an energy-dispersive X-ray spectroscopy (EDS, Oxford). Evolution of surface roughness of the pure Zn after immersion were investigated by a scanning whitelight interferometric (SWLI, Bruker ContourGT-K) with a 0.1 nm vertical resolution. The imaged area is 127 × 95 μm^2^ and is displayed in 3D profile. The chemical states of corrosion products were evaluated by X-ray photoelectron spectroscopy (XPS, PHI 5600) with Al Kα radiation (1486.6 eV). The high-resolution spectra were acquired at 55 eV for passage of the species of interest, such as carbon and zinc. The data were analyzed with Xpspeak 41 software after energy calibration. The reference peak was the C1s at 284.5 eV. In addition, the phase composition of the corrosion products was analysed by X-ray diffractometer (XRD, Rigaku) with a Cu target. The diffraction patterns were obtained between 2*θ* values of 10–90° at a scanning rate of 2° min^−1^.

### Electrochemical measurements

Electrochemical measurements were performed by an electrochemical analyser (ModuLab XM). A three-electrode cell set-up was used wherein the pure Zn samples, saturated calomel electrode (SCE) and a platinum sheet were used as the working, reference and counter electrodes, respectively. For the working electrode, the backside of the square samples was connected to a copper wire and then sealed with epoxy to expose the research surface area of 1 cm^2^. All the samples were immersed in the solutions for different times. A water bath was used to keep the solution temperature at 37 ± 0.5 ^o^C. The experiments were conducted in aerated conditions.

Potentiodynamic polarization (PDP) tests were started at a potential 0.2 V below the OCP and moved in the anodic direction to −0.60 V vs. SCE at a constant scan rate of 1 mV s^−1^. Electrochemical impedance spectroscopy (EIS) studies were carried out at OCP after various immersion times (ranged from 0.5 to 336 h) for the same sample. The experiments were performed at 5 mV sinusoidal amplitude in the frequency range of 10 to 10^−1^ Hz. Each test was done on three replications for studying the repeatability of the results. The impedance data were analysed with the ZSimpWin software package and fitted to the equivalent curves.

## Results

### Effects of BSA concentration on electrochemical behaviour of the pure Zn

**Fig. 1:**
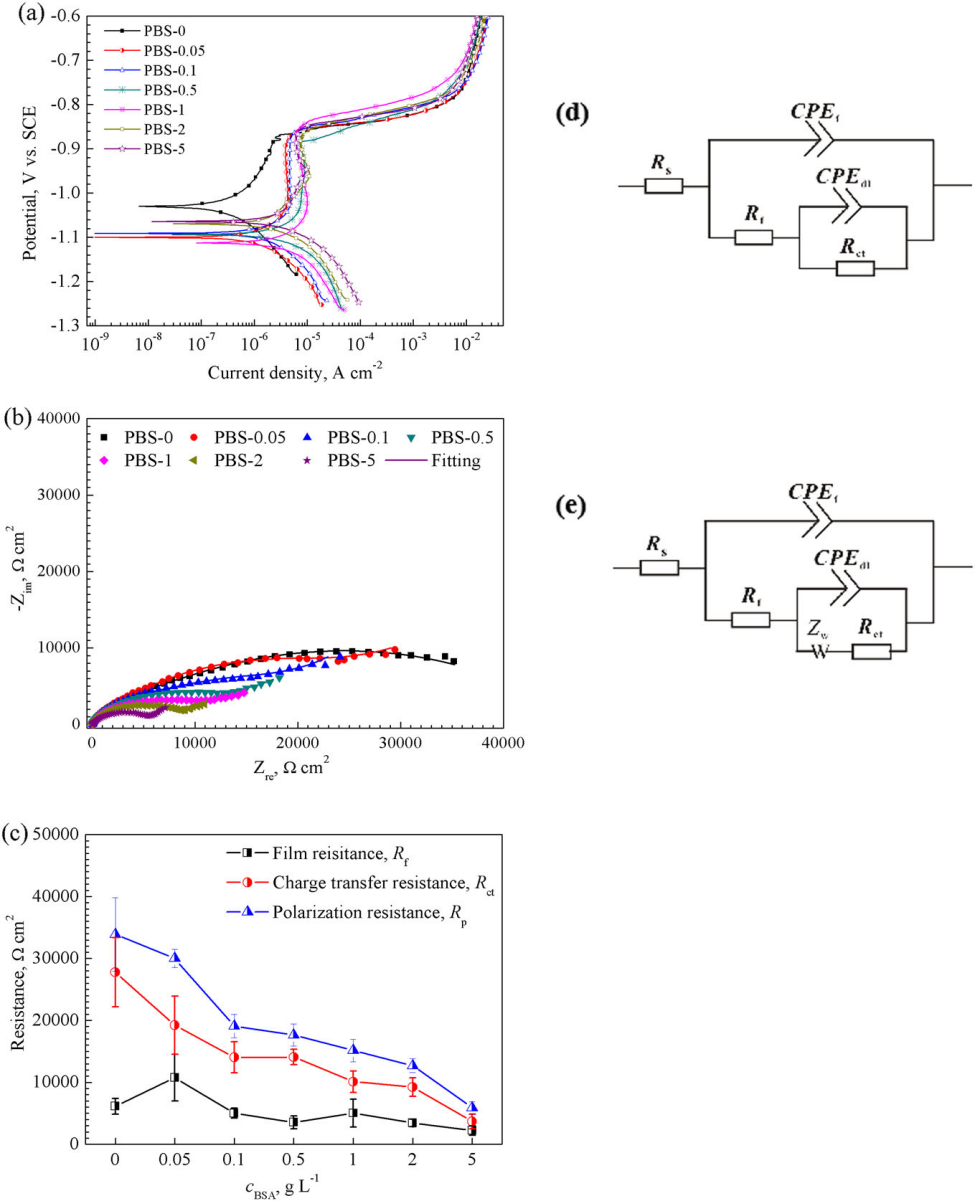
**a** Potentiodynamic polarization curves and **b** Nyquist plots of pure Zn in PBS containing BSA of 0 g L^−1^, 0.05 g L^−1^, 0.1 g L^−1^, 1 g L^−1^, 2 g L^−1^, 5 g L^−1^ (donated as PBS-0, PBS-0.05, PBS-0.1, PBS-1, PBS-2, and PBS-5 in text); **c** the fitted resistances; **d** and **e** the equivalent circuits used for EIS data: **d** is used to fit the EIS data obtained in PBS-0 at all intervals, in PBS-0.1 at 5 h and 24 h, in PBS-2 at 5 h and after 168 h, **e** is used to the EIS obtained in PBS-0.1 for 0.5 h and after 48 h, in PBS-2 for 0.5, 24, and 48 h

The polarization curves of pure Zn were obtained in PBS with various BSA concentrations (0, 0.05, 0.1, 0.5, 1, 2, and 5 g L^−1^) after 0.5 h immersion. Figure 1a presents the anodic and cathodic polarization behaviour of the pure Zn under different BSA concentrations. No obvious linear Tafel region was observed in the anodic branch. The values of corrosion potential (*E*_corr_) and corrosion current density (*i*_corr_) were obtained by the interpolation of cathodic Tafel slopes (*β*_c_) [39, 40]. The intersection of the cathodic asymptote and the line crossing the *E*_corr_ has been introduced as the corrosion current density *i*_corr_ (Fig. S2). Related electrochemical parameters were collected and listed in Table 1. The *E*_corr_ value of pure Zn is marginally lowered by addition of BSA compared to that in PBS-0, which indicates an enhancement of the sensitivity to corrosion of pure Zn. The *i*_corr_ value progressively rises from 0.42 ± 0.03 μA cm^−2^ for PBS-0 to 7.65 ± 0.12 μA cm^−2^ when the BSA concentration increases to 5 g L^−1^. For the anodic branch, a passivation-like behaviour appears for the samples in BSA containing solutions, denoting that a film provisionally forms on the surface and impedes pitting corrosion. The breakdown potentials are around −0.87 V vs. SCE, beyond which a sharp increase in the anodic current density occurs. The current density in the passive-like region, *i*_pass_ obtained at a potential of −0.92 V vs. SCE is much higher than that in PBS-0 (1.63 μA cm^−2^). And the *i*_pass_ value rises with BSA concentration from 3.95 μA cm^−2^ for PBS-0.05 to 7.14 μA cm^−2^ for PBS-5. At the case of concentration beyond 1 g L^−1^, the passivation becomes more apparent as the current density reduces moderately with the increase of potential. And even a brief peak around −0.95 V vs. SCE is unveiled in PBS-2 and PBS-5, beyond which the current density is suppressed obviously. As for the cathodic part, similar shapes of all the curves indicate the same reaction in the cathodic area. The cathodic current density also displays an increment with BSA concentration. In comparison, the presence of BSA shifts both the anodic and cathodic current densities, signifying an enhancement in the pure Zn dissolution and water reduction by the BSA [14].

**Table 1:** Electrochemical parameters obtained from PDP curves and fitting data of EIS for the pure Zn after immersion for 0.5 h in PBS with different concentrations of BSA.(±) is attributed to the scatter band corresponds to the maximum and minimum values around the mean values of three replicates

	PDP		EIS							
Solutions	*E*_corr_ (V/SCE)	*i*_corr_ (µA cm^−2^)	CPE_f_ (10^−5^ Ω^−1^ cm^−2^ s^n1^)	*n*_1_	*R*_f_ (kΩ cm^2^)	CPE_dl_ (10^−5^ Ω^−1^ cm^−2^ s^n2^)	*n*_2_	*R*_ct_ (kΩ cm^2^)	*W* (10^−3^ Ω^−1^ cm^−2^ s^0.5^)	*χ*^2^ (10^−3^)
PBS-0	−1.01 ± 0.01	0.42 ± 0.03	0.94 ± 0.02	0.80 ± 0.01	6.13 ± 1.26	0.61 ± 0.15	0.50 ± 0.05	27.78 ± 5.57	−	1.28 ± 0.19
PBS-0.05	−1.11 ± 0.01	1.35 ± 0.27	1.37 ± 0.30	0.78 ± 0.03	10.77 ± 3.77	5.87 ± 1.89	0.76 ± 0.10	19.23 ± 4.69	0.52 ± 0.40	2.04 ± 0.51
PBS-0.1	−1.09 ± 0.01	1.86 ± 0.11	1.32 ± 0.12	0.80 ± 0.02	5.02 ± 0.81	6.21 ± 0.30	0.59 ± 0.04	14.05 ± 2.50	0.56 ± 0.02	1.15 ± 0.24
PBS-0.5	−1.09 ± 0.01	3.28 ± 0.36	1.35 ± 0.09	0.82 ± 0.01	3.55 ± 1.04	5.48 ± 0.82	0.46 ± 0.02	14.09 ± 1.24	0.62 ± 0.12	0.90 ± 0.05
PBS-1	−1.11 ± 0.01	3.26 ± 0.80	1.51 ± 0.26	0.80 ± 0.03	5.03 ± 2.24	8.42 ± 3.96	0.60 ± 0.13	10.11 ± 1.73	0.60 ± 0.25	1.43 ± 0.48
PBS-2	−1.06 ± 0.01	4.85 ± 0.26	1.42 ± 0.37	0.80 ± 0.03	3.45 ± 0.58	6.46 ± 1.36	0.58 ± 0.07	9.24 ± 1.50	1.17 ± 0.42	1.05 ± 0.35
PBS-5	−1.06 ± 0.01	7.65 ± 0.12	1.54 ± 0.23	0.80 ± 0.02	2.22 ± 0.76	7.26 ± 2.09	0.63 ± 0.11	3.68 ± 1.17	1.51 ± 0.19	1.25 ± 0.43

From the Nyquist plots (Fig. 1b), two capacitive loops are observed for the sample in PBS-0. With the addition of BSA, the diameter of the capacitive loop gradually diminishes and a Warburg impedance character emerges in the low frequency region. In the Bode plots (Fig. S3), two overlapped time constants can be distinguished. The impedance modulus |Z| at the lowest frequency shows an incline trend with BSA concentrations. In view of the pores in BSA adsorption film or the corrosion products, the electrolyte subsequently reacts at the pure Zn surface [41], a two-time constant model is used to fit the experimental EIS data of pure Zn. As shown in Fig. 1d, *R*_s_ represents the solution resistance, *R*_f_ and *CPE*_f_ describe the first capacity loop at medium frequency, which represent the film resistance and capacity, respectively. *R*_ct_ and *CPE*_dl_ are used to describe the second capacity loop at low frequency, which represents the charge transfer resistance and the electric double layer at the metal/electrolyte interface. CPE is a constant phase element and is employed to account for the non-ideal behaviour of the capacity elements in the system, such as surface heterogeneity [41]. The electrical impedance of a CPE can be represented by Eq. (1):

1 $$Z_{{{{{{\mathrm{CPE}}}}}}} = \frac{1}{{(j\omega )^nY_0}}$$

where *Y*_0_ is the general admittance function with unit of F cm^−2^ S^(*n*−1)^, *ω* represents the angular frequency and *n* is a coefficient related to dispersive behaviour with values between 1.0 and 0. A value of 1 relates to a capacitor, a value of 0 is characteristic for a resistor and 0.5 corresponds to diffusion behaviour [42]. As for the BSA containing solutions, a diffusion behaviour emerges in the low frequency. Thus a two-time constant model with the Warburg impedance (Fig. 1e) is used to fit the EIS data obtained from these solutions. Corresponding electrical parameters are presented in Table 1. The chi-square values (*χ*^2^) are less than 2.10 × 10^−3^, indicating a satisfactory fit. The simulated values of *R*_f_, *R*_ct_, and *R*_p_ are summarized in Fig. 1c.

### Effects of immersion time on electrochemical behaviour of the pure Zn

**Fig. 2:**
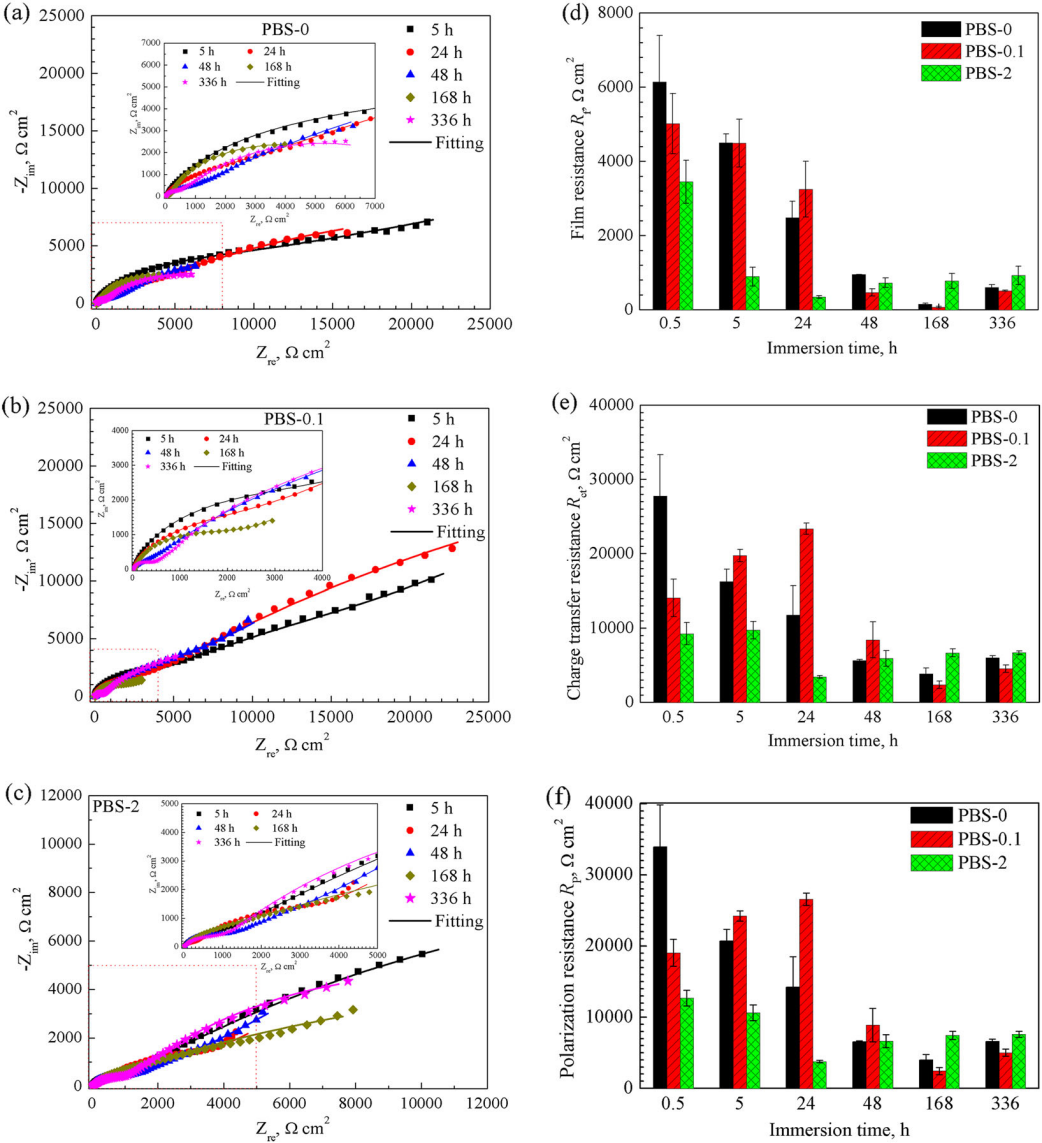
Nyquist plots, **a** surface film resistance *R*_f_, **b** charge transfer resistance *R*_ct_, and **c** total resistance *R*_p_ of the pure Zn immersed in PBS-0, PBS-0.1, PBS-2 for different duration. Insertion in **a–c** portrays the selected portion of the spectra

The EIS was performed on the pure Zn after immersion in PBS-0, PBS-0.1, and PBS-2 to investigate the effect of immersion duration. The Nyquist plots in Fig. 2a–c show two-time constants in three selected solutions. One high frequency capacity loop and one low frequency capacity loop correspond to the characteristic of corrosion product film and electric double layer, respectively. Dimension of the loop in PBS-0 dwindles with immersion time, which is perceived clearly from the values of impedance modulus |Z| (Fig. S4a). For the sample in PBS-2, the capacity loop dimension shrinks within 24 h, and gradually increases after 48 h (Fig. 2c). In PBS-0.1, the phase angles of pure Zn after immersion for 48 h approach 45° (Fig. S4b), denoting mass transfer at the metal/electrolyte interfaces. Similar phenomenon can be found in PBS-2 after 24 and 48 h (Fig. S4c). The EEC model with a Warburg impedance in Fig. 2e is used to fit the EIS data obtained in PBS-0.1 after 48 h and PBS-2 for 24 h, 48 h. EIS data at other points are fitted by EEC model in Fig. 2d. Table 2 displays corresponding parameters. The film resistance *R*_f_, charge transfer resistance *R*_ct_ and polarization resistance *R*_p_ obtained from the sum of *R*_f_ and *R*_ct_ are extracted in Fig. 2d–f. The *R*_p_ values in PBS-0 decline with immersion time (before 168 h) and a trivial increase appears at 336 h, which indicates the corrosion of pure Zn is encouraged with time in PBS-0. However, the *R*_p_ value in PBS-0.1 increases within the first 24 h and falls sharply from 26.55 to 8.88 kΩ after 48 h. The lowest value is obtained at 168 h, after which the *R*_p_ mildly rises again. The *R*_p_ in PBS-2 shows a declined tendency within the first 24 h and sustains at a bit higher level after 48 h. Combined with EIS plots, we speculate that at the early stage of immersion (24 h), the corrosion of pure Zn is hampered by a low concentration (<0.1 g L^−1^) of BSA, while higher concentration (i.e. 2 g L^−1^) leads to a higher corrosion rate. With time elapses, influence of BSA on pure Zn corrosion become smaller after 48 h.

**Table 2:** Fitting data of EIS for the pure Zn in PBS-0, PBS-0.1, and PBS-2 after immersion for 5, 24, 48, 168, and 336 h. (±) is attributed to the scatter band corresponds to the maximum and minimum values around the mean values of three replicates

Solution	Immersion time	CPE_f_ (10^−6^ Ω^−1^ cm^−2^ s^n1^)	*n*_1_	*R*_f_ (kΩ cm^2^)	CPE_dl_ (10^−4^ Ω^−1^ cm^−2^ s^n2^)	*n*_2_	*R*_ct_ (kΩ cm^2^)	*W* (10^−3^ Ω^−1^ cm^−2^ s^0.5^)	*χ*^2^ (10^−3^)
PBS-0	5 h	10.63 ± 1.30	0.78 ± 0.02	4.50 ± 0.24	1.05 ± 0.16	0.41 ± 0.03	16.24 ± 1.68		1.13 ± 0.47
	24 h	12.92 ± 3.02	0.77 ± 0.01	2.48 ± 0.45	1.68 ± 0.75	0.36 ± 0.05	11.78 ± 3.92		1.04 ± 0.18
	48 h	22.15 ± 3.55	0.77 ± 0.01	0.95 ± 0.00	4.01 ± 0.24	0.40 ± 0.02	5.61 ± 0.13		1.47 ± 0.17
	168 h	65.52 ± 20.83	0.77 ± 0.05	0.15 ± 0.00	6.32 ± 1.60	0.76 ± 0.04	3.84 ± 0.78		1.29 ± 0.25
	336 h	18.82 ± 0.69	0.86 ± 0.01	0.60 ± 0.01	4.19 ± 0.31	0.59 ± 0.01	6.01 ± 0.29		1.73 ± 0.50
PBS-0.1	5 h	5.92 ± 0.35	0.85 ± 0.01	4.49 ± 0.65	0.96 ± 0.04	0.51 ± 0.03	19.74 ± 0.83		1.66 ± 0.33
	24 h	7.19 ± 1.11	0.84 ± 0.01	3.25 ± 0.75	0.82 ± 0.28	0.56 ± 0.05	23.3 ± 0.77		1.39 ± 0.04
	48 h	29.39 ± 4.77	0.65 ± 0.13	0.47 ± 0.09	1.02 ± 0.51	0.68 ± 0.16	8.41 ± 2.42	0.43 ± 0.04	1.59 ± 0.42
	168 h	86.86 ± 8.44	0.78 ± 0.11	0.07 ± 0.02	4.32 ± 1.22	0.83 ± 0.04	2.37 ± 0.50	2.19 ± 0.33	1.90 ± 0.73
	336 h	31.80 ± 20.5	0.81 ± 0.09	0.51 ± 0.01	5.48 ± 1.50	0.73 ± 0.10	4.52 ± 0.50	1.40 ± 0.36	2.14 ± 0.66
PBS-2	5 h	7.37 ± 0.88	0.83 ± 0	0.89 ± 0.25	1.84 ± 0.40	0.47 ± 0.05	9.73 ± 1.18		0.78 ± 0.03
	24 h	14.90 ± 1.95	0.79 ± 0.01	0.34 ± 0.04	1.76 ± 0.31	0.65 ± 0.02	3.43 ± 0.20	1.44 ± 0.44	0.71 ± 0.03
	48 h	9.20 ± 2.34	0.83 ± 0.03	0.73 ± 0.13	2.48 ± 1.58	0.45 ± 0.08	5.91 ± 1.06	1.47 ± 0.84	0.80 ± 0.12
	168 h	31.55 ± 15.23	0.70 ± 0.03	0.78 ± 0.20	3.39 ± 0.82	0.38 ± 0.04	6.66 ± 0.54		0.49 ± 0.09
	336 h	33.46 ± 16.89	0.71 ± 0.03	0.92 ± 0.25	6.06 ± 1.99	0.61 ± 0.05	6.78 ± 0.25		1.75 ± 1.29

### Micromorphology and component analysis

No obvious difference was observed on morphology of the pure Zn after immersion for 0.5 h in different solutions (shown in Fig. S5). Some slight scratches only during polishing are still detected by SEM. After immersion for 0.5 h, the general elements of Zn, C, O, P, and Na are detected on all samples, while element N is only observed for samples immersed in BSA containing solutions (Table S2). Element P comes from phosphate in the solution and the atomic ratio P/Zn is used to character the adsorbed PO_4_^3-^ after immersion. The value of P/Zn is decreased from 0.013 to 0.007 and N/Zn is increased to 0.064 by addition of 0.05 g L^−1^ BSA. However, further increase of BSA makes the contents of P and N change irregularly.

**Fig. 3:**
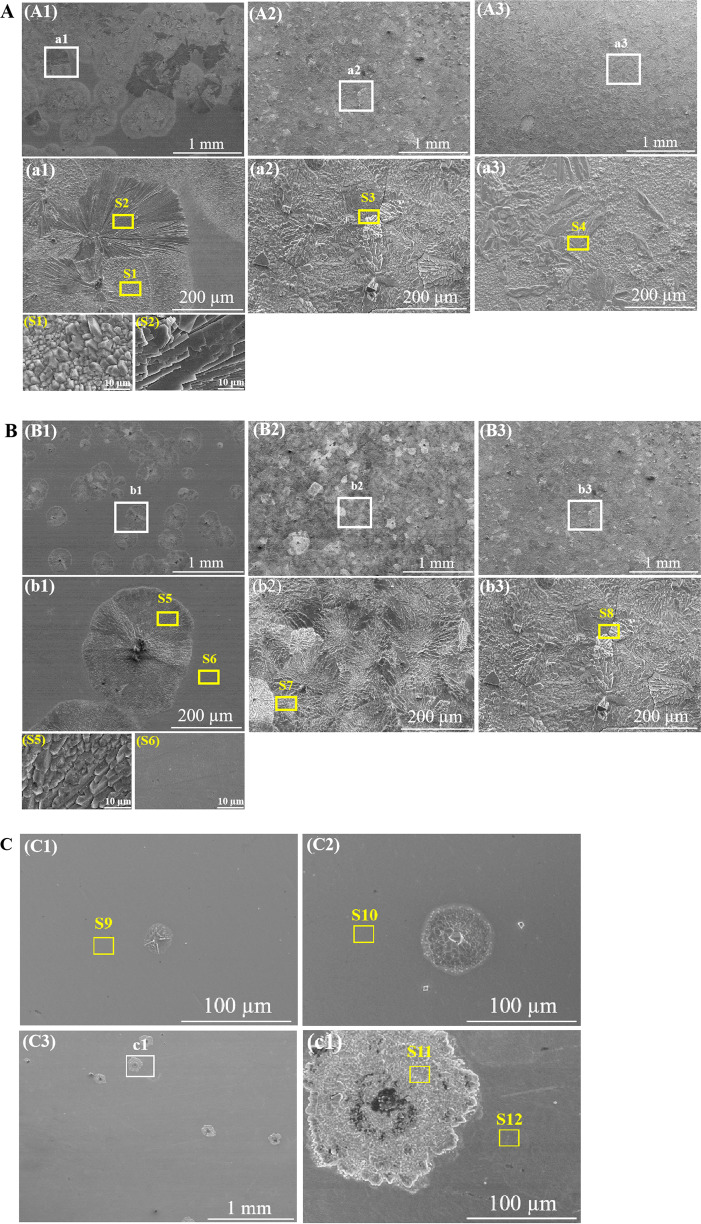
SEM images of pure Zn after immersion in **A** PBS-0, **B** PBS-0.1 and (PBS-2) for (A1, B1, C1) 48 h, (A2, B2, C2) 168 h and (A3, B3, C3) 336 h; Yellow squares in S1-S10 indicate the area for EDS analysis

The morphology of pure Zn immersed in PBS-0 for different intervals are depicted in Fig. 3A. It is notable that round flower-like corrosion products account for most of the sample surface after immersion for 48 h (Fig. 3A1). In the high magnifications, these flowers are comprised of prism (Fig. 3S1) and sheet (Fig. 3AS2) structures. With the immersion time extends to 168 h, the number of round “flowers” increases and finally they emerge together (Fig. 3AA2). The whole sample surfaces are entirely covered by the corrosion product layer with some agglomerates (Fig. 3Aa2), which become more evident after 336 h (Fig. 3AA3 and 3a3).

In the presence of 0.1 g L^−1^ BSA, the size and number of round flower-like corrosion products are significantly reduced in the first 48 h (Fig. 3AB1 and b1). Further inspection reveals that the corrosion products are mainly comprised of prism structures (Fig. 3AS5). After 168 h, the number of round flowers is obviously increased and the corrosion products uniformly cover the surface (Fig. 3AB2 and 3b2). When the immersion time is prolonged, some crystal aggregations of the corrosion products are presented on the surface (Fig. 3AB3 and b3).

In the case of addition of 2 g L^−1^ BSA, the sample surface is relatively smooth in the first 48 h (Fig. 3AC1). After 168 h, the size of the “round flower” is enlarged. And the appearance of the crystal totally changes to polyhedron (Fig. 3AC2). After 336 h, sparse corrosion product crystals emerge on the surface (Fig. 3AC3).

Table 3 shows the EDS analysis for the pure Zn immersed in various solutions for different durations. The predominant component formed on the pure Zn is C, Zn, O, Na, P. The C might be related to adventitious carbon, coming from contamination during exposure to the air for the samples in PBS. The N, detected on the samples in PBS-0.1 and PBS-2 corresponds to the BSA adsorption. The atomic ratio of Zn/Na/P approaches 1/1/1 for the prismatic structures, which is close to the stoichiometric of sodium zinc phosphate.

**Table 3:** Elemental compositions of pure Zn surfaces after immersion in different solutions for different time (Corresponding areas were indicated in Figs. 8–10 by yellow squares). Data were expressed as mean ± standard deviation

Solution	Immersion time	Area	C	O	Zn	Na	P	K	N
PBS-0	48 h	S1	26.6 ± 3.6	45.3 ± 2.1	9.6 ± 0.7	9.9 ± 0.9	8.1 ± 0.3	0.3 ± 0.0	–
		S2	16.9 ± 0.3	51.9 ± 1.1	17.9 ± 0.6	5.0 ± 0.2	8.5 ± 0.3	–	–
	168 h	S3	20.8 ± 0.3	49.4 ± 0.9	10.7 ± 1.2	10.8 ± 0.8	7.8 ± 0.2	0.4 ± 0.0	–
	336 h	S4	11.5 ± 1.3	48.2 ± 2.1	14.6 ± 0.4	14.3 ± 0.6	11.0 ± 0.8	0.3 ± 0.1	–
PBS-0.1	48 h	S5	13.0 ± 2.1	52.4 ± 1.6	12.8 ± 0.9	12.8 ± 1.2	9.0 ± 0.5	–	–
		S6	39.2 ± 1.2	14.5 ± 0.5	43.9 ± 2.1	–	1.2 ± 0.1	–	0.9 ± 0.1
	168 h	S7	16.7 ± 2.1	50.4 ± 1.5	12.1 ± 1.5	11.4 ± 0.8	9.2 ± 0.3	0.3 ± 0.1	–
	336 h	S8	10.0 ± 0.6	51.9 ± 1.3	13.1 ± 0.6	14.5 ± 0.5	10.3 ± 1.3	0.3 ± 0.0	–
PBS-2	48 h	S9	36.3 ± 1.1	11.6 ± 0.2	49.8 ± 0.1	–	0.7 ± 0.1	–	1.5 ± 0.1
	168 h	S10	34.2 ± 1.1	21.3 ± 1.3	39.8 ± 0.6	–	2.8 ± 0.2	–	2.6 ± 0.1
	336 h	S11	28.4 ± 5.6	33.8 ± 3.2	17.3 ± 1.0	8.4 ± 2.1	10.9 ± 1.1	0.3 ± 0.3	0.9 ± 0.3
		S12	34.2 ± 0.6	26.8 ± 2.3	35.3 ± 0.3	–	3.5 ± 1.2	–	2.7 ± 0.3

– indicates no detection of such elements

**Fig. 4:**
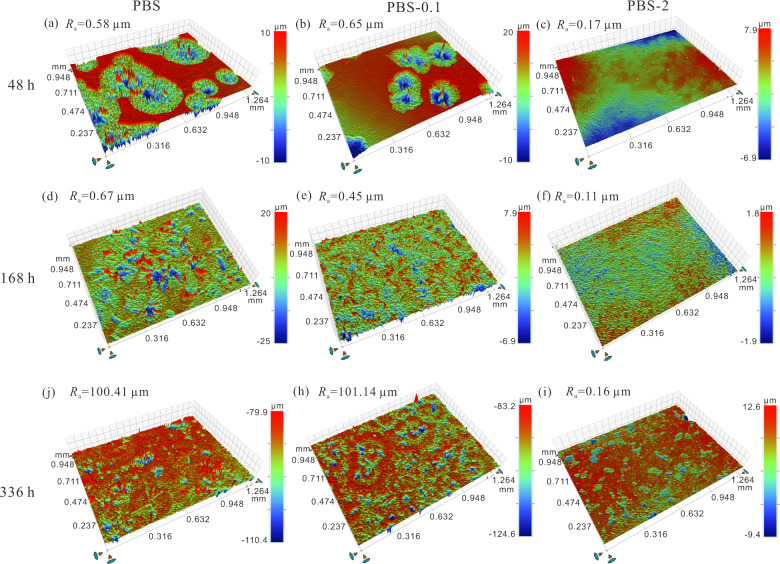
3D profile images of the pure Zn after immersion in **a, d, g** PBS-0, **b, e, h** PBS-0.1 and **c, f, i** PBS-2 for **a**–**c** 48 h, **d–f** 168 h, and **g–i** 336 h

3D surface profile images of the samples measured using SWLI are shown in Fig. 4. After 48 h, the sample in PBS-0 shows the largest corrosion area with an average surface roughness of *R*_a_ = 0.58 μm (Fig. 4a). Whereas in PBS-2, the sample surface is relatively smooth with the lowest value of *R*_a_ = 0.17 μm (Fig. 4c). Visible localized corrosion in PBS-0.1 give rise to the biggest roughness of *R*_a_ = 0.65 μm (Fig. 4b). With time elapses, pure Zn corrosion is propelled unceasingly and finally corrosion products occupy the sample surfaces immersed in PBS-0 and PBS-0.1 after 168 h, which leads to a minor change in the roughness (Fig. 4d and e). After longer immersion time, the surface roughness sharply increases to *R*_a_ = 100.41 μm for PBS-0 and *R*_a_ = 101.14 μm for PBS-0.1 (Fig. 4g, h), which may be caused by the corrosion products aggregation as observed in Fig. 3A3 and 3B3. For the samples in PBS-2, surface roughness merely changes a little throughout the test because of minute amounts of visible corrosion products (Fig. 4f and i).

**Fig. 5:**
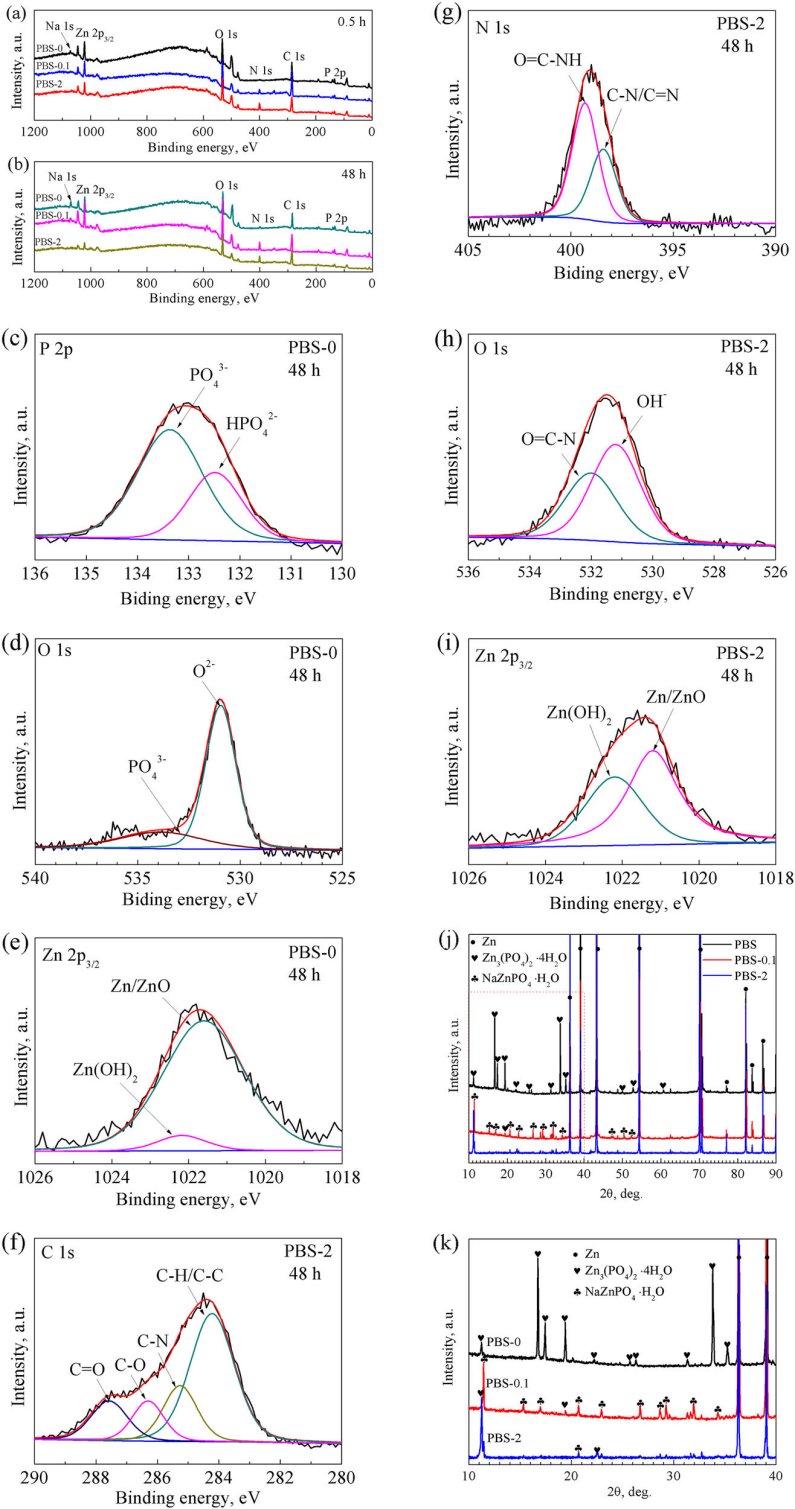
XPS survey of pure Zn after immersion in PBS, PBS-0.1 and PBS-2 for **a** 0.5 h and **b** 48 h; High resolution XPS spectra of **c** P 2p, **d, h** O 1 s, **e, i** Zn 2p_3/2_, **f** C 1s, **g** N 1 s after immersion in **c–e** PBS-0 and **f–i** PBS-2 for 48 h; **j** XRD patterns of the pure Zn after immersion in PBS, PBS-0.1and PBS-2 for 48 h, **k** XRD patterns in the range of 10–40°

XPS spectra of the pure Zn after soaking for 0.5 and 48 h are presented in Fig. 5. The peaks for Na 1s, Zn 2p, O 1s, C 1s, and P 2p are detected on the sample immersed in PBS-0 for 0.5 and 48 h. A signal for N 1s is observed after addition of BSA into the solution (Fig. 5a). And with the immersion, the intensity of N 1 s peak becomes stronger (Fig. 5b), which can be confirmed by the elemental analysis as shown in Table S3. After immersion for 48 h, the atomic percentage (at%) of N on the samples in PBS-0.1 and PBS-2 increase to 10.72 and 10.83, respectively. More notably, the content of P is evidently decreased by the presence of BSA in the first 0.5 h. After 48 h, the P content for the sample in PBS-0.1 is slightly lower than that in the PBS-0, and 2 g L^−1^ BSA results in the lowest P content. The result is consistent with the EDS. A speculation can be made that addition of BSA into the PBS hinders deposition of phosphates on the pure Zn.

Figure 5c–i displays the high-resolution XPS spectra after immersion in PBS-0 and PBS-2 for 48 h. The P-2p peak (Fig. 5c) is fitted with two contributions at 133.6 and 132.6 eV, which correspond to PO_4_^3-^ and HPO_4_^2-^, respectively [43, 44]. These two peaks account for the formation of phosphates on the sample surface. With respect to O-1s spectra (Fig. 5d), the peak is composed of two contributions at 530.1 eV and 533.85 eV, which represent O^2-^ and PO_4_^3-^ group [45, 46]. The deconvolution of Zn-2p_3/2_ spectra (Fig. 5e) reveals two components of Zn/ZnO and Zn(OH)_2_, which are found at 1021.6 and 1022.7 eV [47].

As for the sample in PBS-2, the peak for C-1s are fitted with four contributions (Fig. 5f). Generally, the signal at 284.2 eV is assigned to C-H/C-C bonds. The peak at 286.3 eV represents the C-O group of the protein backbone [48, 49]. And the signals at 285.2 and 287.5 eV correspond to C–N and C=O, respectively. The characteristic bond suggests the presence of BSA on the sample surface. Figure 5g displays the deconvolution of N 1s spectra. The signals centred at 398.4 and 399.3 eV are assigned to the C-N and C=O-NH bonds, which come from amine or amide groups in the BSA [50, 51]. As exhibited in Fig. 5h, O-1s spectra is composed of two contributions centred at 531.2 and 532 eV, which represent OH^−^ and O=C-N, respectively. The OH^−^ peak could be originated from hydroxides and hydroxyl groups in the BSA molecule. In addition, the deconvolution of Zn 2p_3/2_ spectra shows the same peaks for Zn/ZnO and Zn(OH)_2_ (Fig. 5i) as aforementioned. The results demonstrate that BSA and ZnO/Zn(OH)_2_ are contained in the corrosion products after immersion in BSA containing solutions for 48 h.

To confirm the component of the corrosion products, XRD analysis is utilized. Figure 5j shows the XRD patterns of pure Zn after immersion in PBS, PBS-0.1, and PBS-2 for 48 h, and the enlarged area of 10–40° is displayed Fig. 5k. Addition to the peaks for Zn, intense peaks for Zn_3_(PO_4_)_2_ are detected on the sample immersed in PBS-0. Chen et al. [2] has reported that after 3 days immersion in PBS, the corrosion products on pure Zn are comprised of Zn_3_(PO_4_)_2_·4H_2_O, Na_6_Zn_6_(PO_4_)_6_·8H_2_O, and KZn_2_(PO_4_)_2_·2H_2_O. In this work, a small amount of potassium is detected in the crystalline corrosion products after immersion in PBS and PBS-0.1 (Table 3), though the potassium containing structure is not detected by XRD. This may by caused the short immersion time that is not enough for potassium to participate in the crystalline corrosion products. But the spectrum of PBS-0.1reveals that the presence of BSA (0.1 g L^−1^) promotes the formation of NaZn(PO_4_)·H_2_O, which can be attributed to the attachment of Na^+^ to the BSA molecules. For the sample in PBS-2, only a few peaks for Zn_3_(PO_4_)_2_ is observed.

## Discussion

### Adsorption of BSA on the pure Zn

BSA as a typical protein model is widely investigated because of the similar amino sequence to human serum albumin [52]. It has been reported that BSA can be adsorbed on solid surfaces through a complex process, including van der Waals, hydrophobic, electrostatic interactions, and hydrogen bonds [53]. Under the neutral physiological condition of pH 7.4, BSA molecule (isoelectric pH 4.5-4.7) undergoes a neutral-acidic transition and becomes negatively charged [32]. The negative carboxyl group (-COO) allows attractive electrostatic interaction with positively charged cations, such as Mg^2+^, Ca^2+^ [54–56]. This finding has been used to explain the adsorption of BSA on the CoCrMo [27], Ti6Al4V [56, 57], stainless steel [58], and magnesium alloy [59].

Adsorption isotherms can give mutual information of metal surface and adsorbed species at a constant temperature mathematically. Langmuir adsorption isotherm is the most popular expression, which has been utilized to describe the adsorption of BSA onto the stainless steel and CoCrMo surfaces [28]. The Langmuir isotherm can be expressed as follows:

2 $${\Gamma} = \frac{{B_{{{{{{\mathrm{ads}}}}}}}{\Gamma}_{{{{{{\mathrm{max}}}}}}}c}}{{{{{{{\mathrm{1 + }}}}}}B_{{{{{{\mathrm{ads}}}}}}}c}}$$

in which *c* (mol cm^−3^) is the equilibrium concentration of BSA in the bulk solution, *Γ* (mol cm^−2^) is the amount of BSA, i.e. surface concentration, *Γ*_max_ (mol cm^−2^) is the maximum value of *Γ*, and the parameter *B*_ads_ (cm^3^ mol^−1^) reflects the affinity of the BSA molecules toward the metal surface. Equation (2) can be rearranged to give:

3 $$\frac{c}{{\Gamma}} = \frac{1}{{B_{{{{{{\mathrm{ads}}}}}}}{\Gamma}_{{{{{{\mathrm{max}}}}}}}}} + \frac{c}{{{\Gamma}_{{{{{{\mathrm{max}}}}}}}}}$$

A plot of *c*/*Γ* versus concentration *c* should yield a straight line with parameters *Γ*_max_ and *B*_ads_ derived from the slope and intercept, respectively. However, it is not easy to directly measure the BSA concentration on the sample surface. According to Omanovic et al. [58], BSA adsorption on the stainless steel increases the metal dissolution in PBS. The polarization resistance and current density both depend on the BSA concentration. Therefore, the corrosion current density after being corrected (*i*_corr,c_= *i*_corr_ − *i*_corr,0_) by the current density recorded in the PBS-0 (*i*_corr,0_) can be correlated to the surface concentration of BSA, *i*_corr,c_∝*Γ*, which could be used to replace the *c* in Eq. (3), and *Γ*_max_ can be replaced by *i*_max_. All the relevant parameters are listed in Table S4. The equation is converted to Eq. (4):

4 $$\frac{c}{{i_{{{{{{\mathrm{corr}}}}}}}}} = \frac{1}{{B_{{{{{{\mathrm{ads}}}}}}}i_{{{{{{\mathrm{max}}}}}}}}} + \frac{c}{{i_{{{{{{\mathrm{max}}}}}}}}}$$

**Fig. 6:**
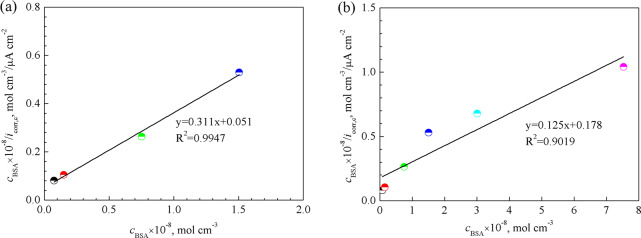
Langmuir adsorption isotherm of BSA on the pure Zn surface. **a** BSA concentration ranges from 0 to 1 g L^−1^ and **b** BSA concentration ranges from 0 to 5 g L^−1^. The unit of BSA concentration (g L^−1^) was transferred into mol cm^−3^

The BSA concentration below 1 g L^−1^ and corresponding current density is fitted in Fig. 6a, while data related to all BSA concentrations is fitted in Fig. 6b. The dependence of *c*/*i*_corr,c_ versus BSA concentration *c* in Eq. (4) is found to be linear in both cases. A high correlation coefficient *R*^2^ = 0.9947 (Fig. 6a) shows that the Langmuir isotherm could be successfully used to describe the adsorption of BSA onto the pure Zn surface when the concentration is below 1 g L^−1^. However, the lower *R*^2^ in Fig. 6b reveals that the high concentration, i.e. 2 and 5 g L^−1^, is not beneficial to BSA monolayer formation. And this is the reason for choice of 0.1 g L^−1^ and 2 g L^−1^ for further longer immersion experiments.

From Eq. (4) and Fig. 6a, a value *i*_max_ (which is proportional to *Γ*_max_) was calculated from the slope of the line to be 320 µA cm^−2^. The intercept yielded *B*_ads_ 6.06 × 10^7^ cm^3^ mol^−1^. The obtained parameter *B*_ads_ is also related to Δ*G*_ads_ (J mol^−1^), the Gibbs free energy of adsorption. The equation is presented as:

5 $$B_{{{{{{\mathrm{ads}}}}}}} = \frac{1}{{c_{{{{{{\mathrm{solvent}}}}}}}}}{{{{{\mathrm{exp}}}}}}\left( {\frac{{ - G_{{{{{{\mathrm{ads}}}}}}}}}{{{{{{{\mathrm{RT}}}}}}}}} \right)$$

where *R* (J mol^−1^ K^−1^) is the gas constant. *T* (K) the temperature, Δ*G*_ads_ (J mol^−1^) the Gibbs free energy of adsorption and the molar concentration of a solvent, which in this case is the water (*c*_H2O_ = 55.5 mol·dm^−3^). According to Eq. (5), the free energy of adsorption of BSA onto the pure Zn surface in PBS is estimated to be around -56.67 kJ mol^−1^ based on the fitting in Fig. 6a. The high value of Δ*G*_ads_ indicates that the BSA molecules can be adsorbed on the pure Zn surface through chemisorption [60].

Although Langmuir isotherm has been utilized to fit the BSA adsorption on some metals, a good fit does not indicate that it is an appropriate model for the adsorption process, i.e. correlation may not imply causation, and the accuracy of the Δ*G*_ads_ value obtained from such data is doubtful [61, 62]. We should verify the exact association between the amount of adsorbed BSA and the current density/resistance of metals in the future. Further study to accurate the free energy of BSA adsorption is in need, especially for the new developed biodegradable metals. And it is possible to reveal an adsorption mechanism of BSA on the molecule level, such as molecule dynamic simulations.

### The effect of BSA concentration and immersion duration

The corrosion of metals is either accelerated by chelation or inhibited by adsorption of BSA [63]. The adsorption competition between PO_4_^3-^ and BSA plays an important role in the metal corrosion, which has been previously studied by other researchers [64, 65]. As is reported [14], the adsorption kinetics of BSA is fast and a steady-state can be reached in a few minutes. Nevertheless, PO_4_^3-^ adsorbed film is supposed to form in a relatively slow process. Preferential adsorption of BSA occupies the active sites on the pure Zn and postpones the PO_4_^3-^ deposition. Therefore, N element appears and lower P content is detected on these sites (Figs. 3S6 and S9) without crystal products (Table 3). However, 0.1 g L^−1^ BSA affects the P content within 48 h only. On the other hand, the contents of Zn, Na, P and O increases with time in the corrosion products in PBS-0 and PBS-0.1, indicating the accumulation of sodium zinc phosphate on the samples.

In addition, BSA diminishes the protection of the adsorption film and speeds up the charge transfer at the interfaces in the initial immersion, which is supported by the decrease of the *R*_f_ and *R*_ct_ of pure Zn at 0.5 h (Fig. 1c). The surface film resistance *R*_f_ is significantly drifted from 6.13 to 10.77 kΩ by the addition of 0.05 g L^−1^ BSA, then decreases mildly with BSA concentration. The surface film shows the best protective performance in PBS-0.05, which suggests that low concentration benefits BSA adsorption to produce a barrier for the metal surface, and increased concentration promotes the corrosion. Combined with the parameters in Table 1, one can see that the addition of BSA impressively decreases the charge transfer resistance *R*_ct_ of the pure Zn. It is possible that the reaction of negatively charged BSA with positively charged Zn^2+^ on the surface favours acceleration of ions release. The values of *CPE*_f_ and *CPE*_dl_ in BSA containing solutions are both higher than those from PBS-0. Wang et al. [14] pointed that the phosphate deposition formed a more compact film, whereas the adsorbed BSA formed a porous film that increased the CPE values. It can be speculated that the adsorbed BSA film cannot protect the metal surface effectively. Overall, the polarization resistance *R*_p_, obtained from the combination of *R*_f_ and *R*_ct_, is stepwise declined with the BSA concentration. Thus, the pure Zn corrosion is promoted by BSA addition in PBS in the initial.

**Fig. 7:**
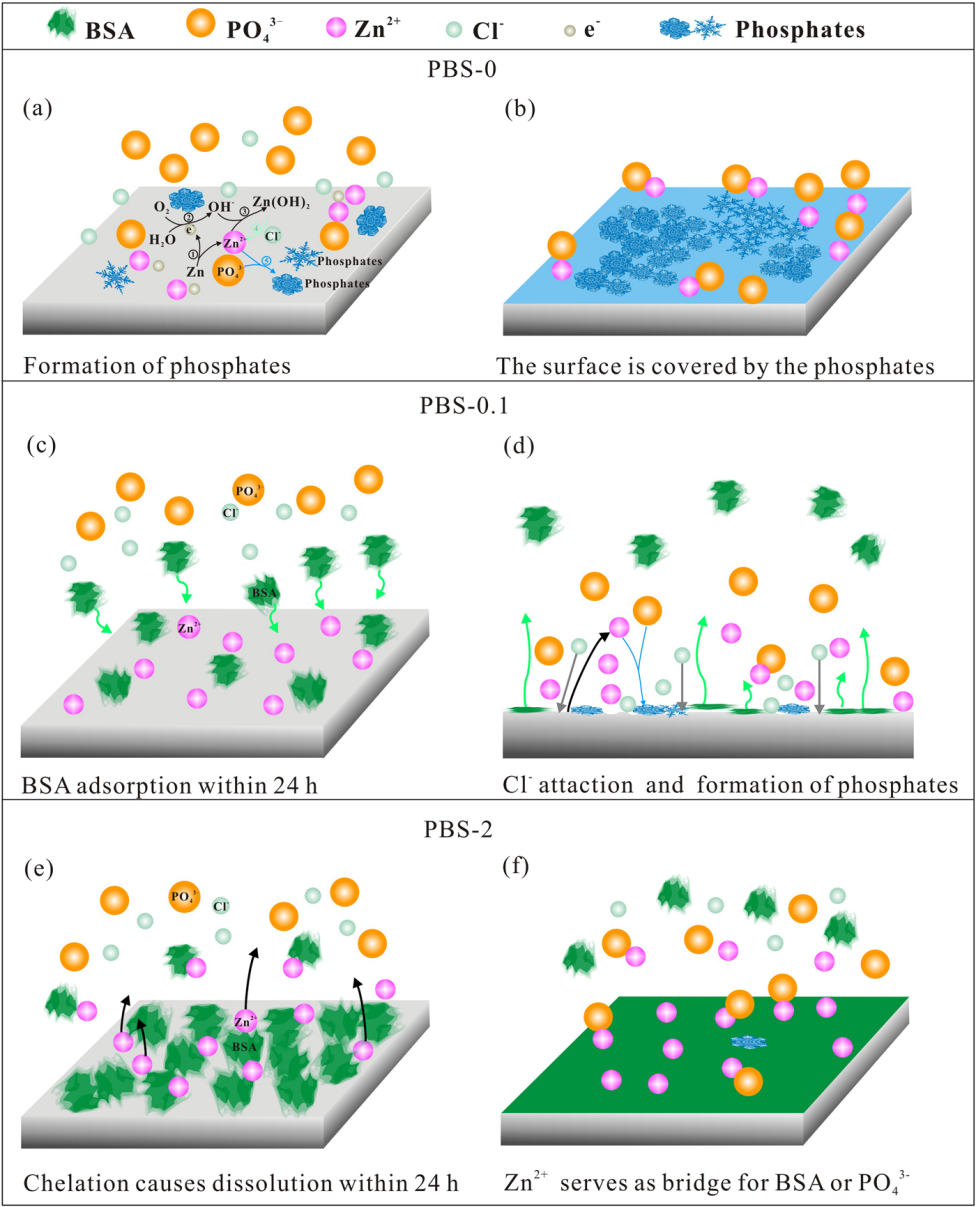
Schematic illustration of interfacial reactions of PBS/BSA and pure Zn surface. **a, b** The process of zinc phosphates formation in the PBS-0; **c, d** initial BSA adsorption and later formation of phosphates in the PBS-0.1; **e, f** chelation of BSA and zinc causes release of Zn, then Zn^2+^ attracts BSA as well as PO_4_^3^

However, influences of BSA concentration on the corrosion are varied after longer immersion time. The proposed schematic corrosion process is exhibited in Fig. 7.

In PBS-0, the corrosion process of pure Zn has been previously demonstrated by Chen et al. [10]. The dissolution of pure Zn and formation of zinc phosphate in the initial stage are presented in Fig. 7a. As time evolves, chloride ions constantly attack the surface, facilitating the damage of the surface. Meanwhile, partially formed phosphates cannot provide sufficient protection for pure Zn. Thus the corrosion is exacerbated within the 168 h (Fig. 1c). With the accumulation of phosphates (Fig. 7b), the corrosion product layer is compact enough to increase the film resistance, and the charge transfer at the interfaces is hindered. As a result, the corrosion resistance of pure Zn is improved after 336 h.

In the presence of BSA, the adsorption layer quickly forms as the metal is exposed to the PBS. Tentatively the adsorbed BSA layer with negatively charges hinders the diffusion and attack of Cl^−^ to the metal surface [66], blocking the reaction 4 and 5 in Fig. 7a. This phenomenon has been found previously on magnesium alloy surfaces by others [51, 67]. The deposition of PO_4_^3-^ is impeded by BSA (Fig. 3B1), leading to a decrease of film resistance in the first 0.5 h. With the time evolves, more and more BSA accumulates on the surface, resulting in an increase in the film resistance as well as the charge transfer resistance within 24 h (Fig. 2d and e). But the BSA concentration of 1 g L^−1^ is too low to cover the whole surface of pure Zn. The Cl^−^ will penetrate the metal through the unoccupied sites, which causes the release of Zn^2+^ ions. The attraction between PO_4_^3-^ and divalent Zn ions promotes the formation of crystalline corrosion products, the size of which continuously increases after the nucleation emerges (Figs. 3B1 and B2). The incomplete BSA layer may gradually peel off from the surface because of the propagation of the metal dissolution (Fig. 7d). It can be seen from the EDS analysis in Table 3, there is little N element on the spots where the crystalline structure forms. Domination of PO_4_^3-^ adsorption after 48 h leads to the accumulation of the phosphates that fully cover the sample surface with time. From then on, the corrosion of pure Zn is barely influenced by BSA (Fig. 2f).

In the case of BSA concentration of 2 g L^−1^, the corrosion resistance of pure Zn is impaired within 24 h. It was reported that in BSA containing solutions the predominant form of the corrosion product of chromium, cobalt and nickel was a metal-BSA complex, with the hydroxides of these metals bound directly with albumin. And the chelation of BSA and surface results in the oxides dissolution and exposure of the underlying metal surface [58]. Herein, competition for the adsorption sites and repulsion between molecules prompts the diffusion of BSA-metal complex (Fig. 7e). The complex of BSA and ZnO/Zn(OH)_2_ on the surface can be accounted for the gradual decrease of film resistance in 24 h. Although fast BSA adsorption can develop a negatively charged barrier that repels anions in the solution to some extent [57]. The BSA layer with pinholes is permeable for ions and water [68, 69]. The released Zn^2+^ in turn acts as bridge for BSA and PO_4_^3-^ adsorption onto the surface (Fig. 7f), which is proved by the simultaneous increased P and N content with time (Table 3). When the adsorption of BSA reaches to an equilibrium, PO_4_^3-^ begins to incorporate with Zn^2+^ to form the deposited phosphates, the size and amount of which are much smaller than that in PBS-0 (Figs. 3C1 and A1). Compared to that in PBS-0, the gradual growth of corrosion product together with the pre-existing BSA layer in PBS-2 retard the pure Zn corrosion after 48 h (Fig. 2f). Combined with the formation of corrosion products, 2 g L^−1^ BSA has more evident effect on the pure Zn corrosion. Higher concentration of BSA can be added in the solution for further exploring the corrosion resistance of pure Zn in the BSA containing solutions.

In this work, PDP and EIS were utilized to study the influence of BSA concentration on the degradation behavior of pure Zn. It should be noted that electrochemical measurements are carried out under applied current, leading to the acceleration of in vitro corrosion. However, the metal in vivo degrades under natural condition, which may vary from the in vitro results. In addition, the concentration and conformation of BSA are different in vivo and in vivo, which could also make a difference in the results. Their influences on pure Zn corrosion will be studied in further work.

## Conclusion

The influence of BSA on the corrosion of pure Zn in PBS were investigated by electrochemical measurements and surface analysis. BSA can change the charge transfer resistance at the zinc/electrolyte interfaces by chemically adsorption. The addition of BSA alters the time-dependent corrosion behaviour of the pure Zn in PBS and either inhibits or accelerates the corrosion depending on the BSA concentrations. At the first 0.5 h, fast adsorption of BSA impedes PO_4_^3-^ deposition and reduces the corrosion resistance of pure Zn. With the time extends, 0.1 g L^−1^ BSA gradually increase the corrosion resistance of the pure Zn within 24 h. And the corrosion is enhanced again after 48 h. The influence of BSA at 2 g L^−1^ shows an opposite trend of the pure Zn corrosion. In addition, formation of phosphates on the pure Zn surface is obstructed by the presence of BSA, especially at concentration of 2 g L^−1^. Overall, 2 g L^−1^ BSA has more evident effect on the pure Zn corrosion.

## Supplementary information

Supplementary Information
