# Supplementary material for: Influence of bovine serum albumin on corrosion behaviour of pure Zn in phosphate buffered saline
Source: J Mater Sci Mater Med. 2021 Aug 18;32(9):95. doi: 10.1007/s10856-021-06567-x (PMC8373726; doi:10.1007/s10856-021-06567-x)
Supplement: Supplementary file 1 — Supplementary Information [file 10856_2021_6567_MOESM1_ESM.pdf]

Supplementary Materials

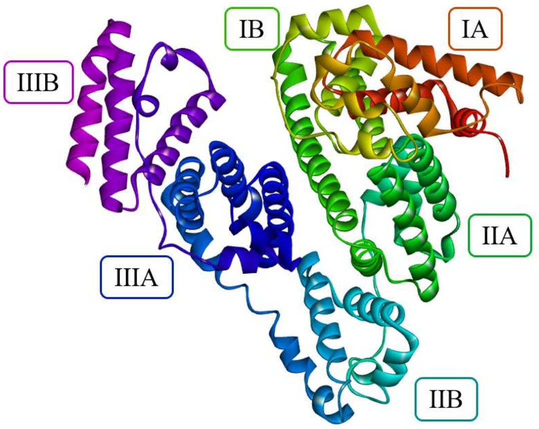

Fig. S1 Schematic of BSA (PDB bank: 4f5s) structure

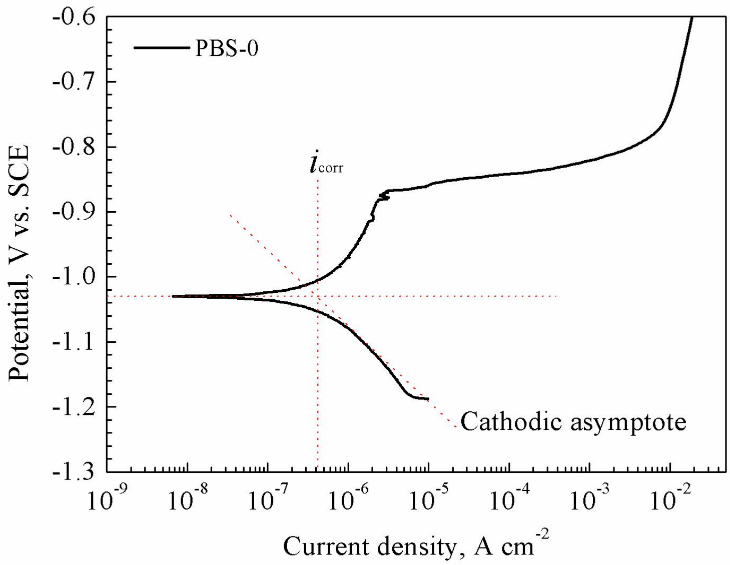

Fig. S2 Typical PDP curve of pure Zn in PBS-0 and the current density obtained by the Tafel extrapolation

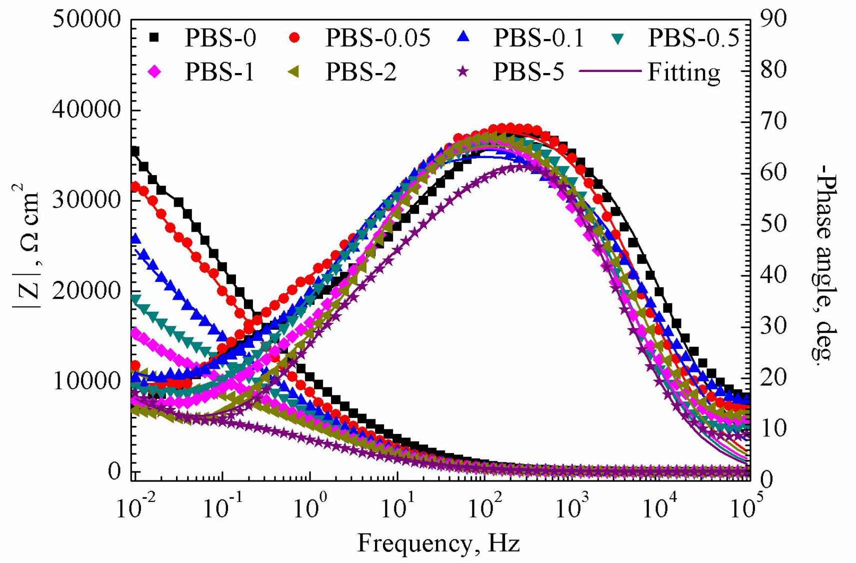

Fig. S3 Bode plots of pure Zn in PBS containing BSA of 0 g·L^{-1}, 0.05 g·L^{-1}, 0.1 g·L^{-1}, 1 g·L^{-1}, 2 g·L^{-1}, 5 g·L^{-1}

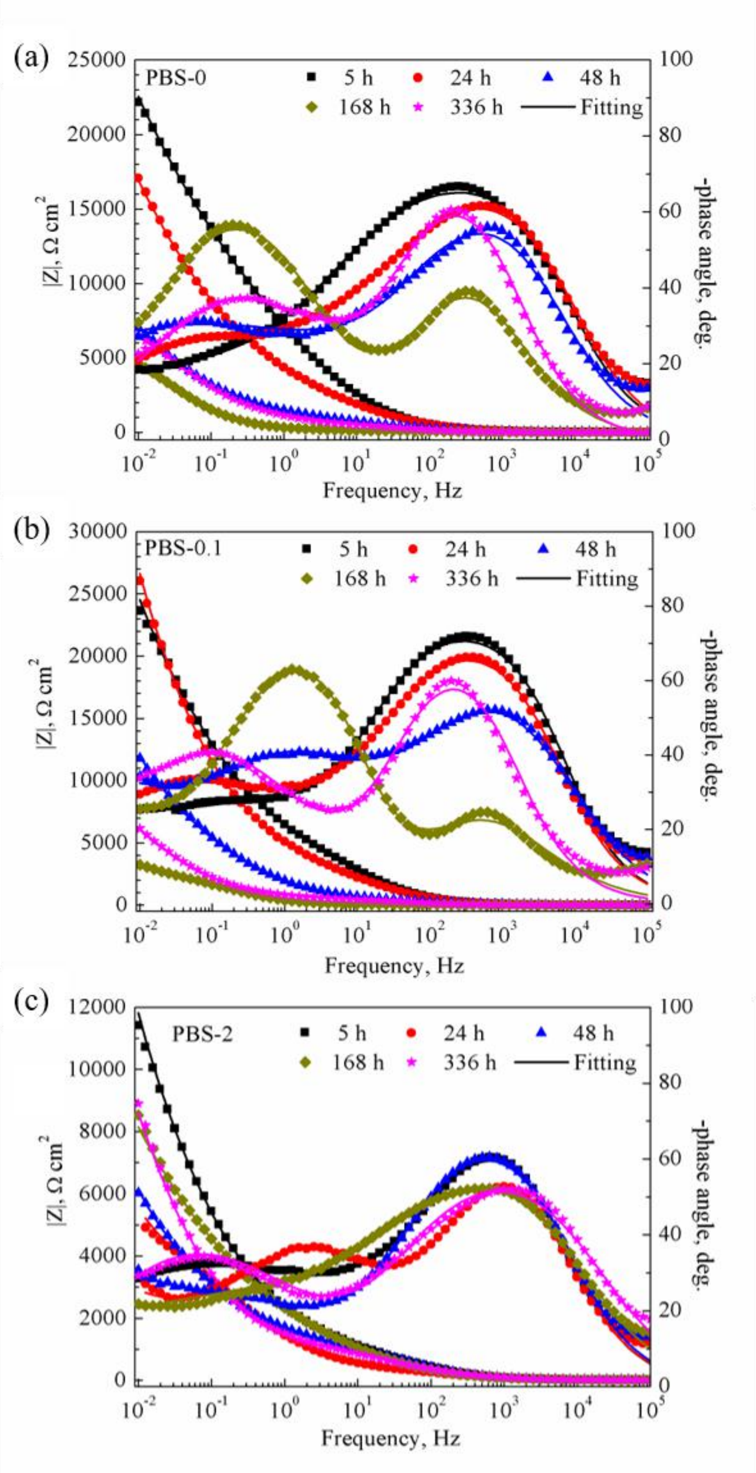

Fig. S4 Bode plots of pure Zn immersed in (a) PBS-0, (b) PBS-0.1 and (c) PBS-2 for different times

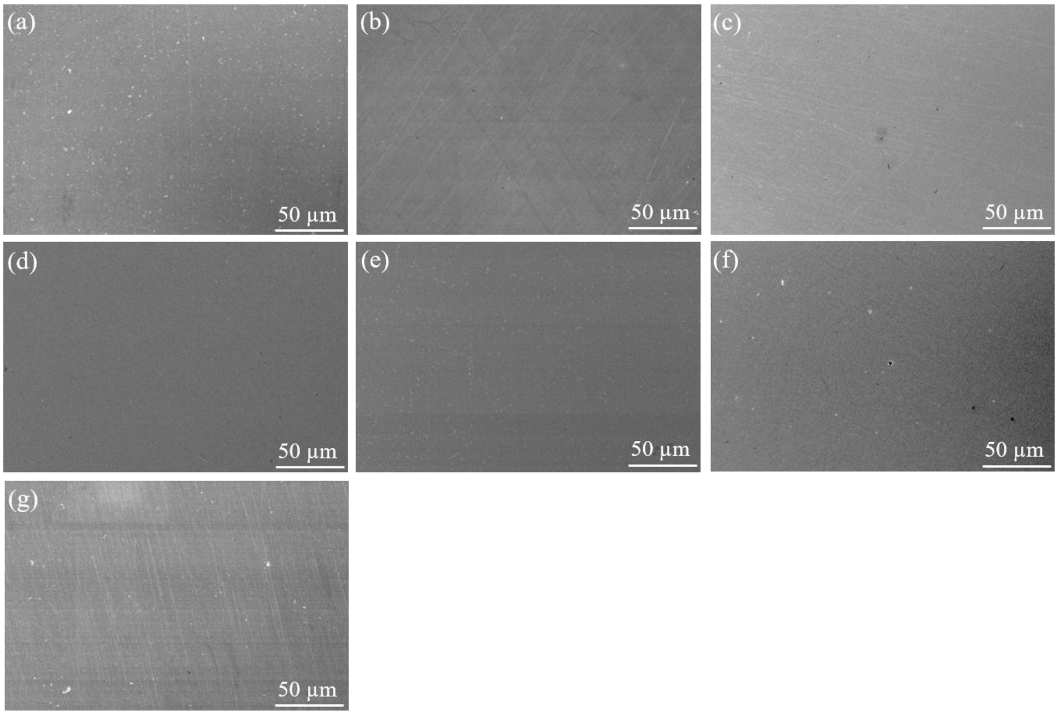

Fig. S5 SEM images of pure Zn in (a) PBS-0, (b) PBS-0.05, (c) PBS-0.1, (d) PBS-0.5, (e) PBS-1, (f) PBS-2 and (g) PBS-5 for 0.5 h

Table S1 Chemical compositions of each solution (g L^{-1}).

Solution ID	NaCl	KCl	Na_2HPO_4	KH_2PO_4	BSA
PBS-0	8.0	0.2	1.15	0.2	0
PBS-0.05	8.0	0.2	1.15	0.2	0.05
PBS-0.1	8.0	0.2	1.15	0.2	0.1
PBS-1	8.0	0.2	1.15	0.2	1
PBS-2	8.0	0.2	1.15	0.2	2
PBS-5	8.0	0.2	1.15	0.2	5

Table S2 Elemental compositions of pure Zn surfaces after immersion in different solutions for 0.5 h. The data were obtained from EDS analysis. (±) is attributed to the scatter band corresponds to the maximum and minimum values around the mean values of three replicates.

Solutions	Zn	C	O	P	Na	N	P/Zn	N/Zn
PBS-0	54.00±3.00	36.07±4.00	7.23±0.76	0.70±0.21	2.10±2.00	0	0.013	0
PBS-0.05	43.00±1.62	46.10±1.57	5.90±0.96	0.30±0.23	2.40±1.95	2.75±0.32	0.007	0.064
PBS-0.1	53.50±3.26	33.20±1.15	9.60±0.51	0.60±0.00	1.60±0.19	1.40±0.85	0.011	0.026
PBS-0.5	50.87±1.22	33.51±4.21	9.93±0.46	0.40±0.01	1.40±0.12	3.17±0.38	0.008	0.062
PBS-1	47.40±1.24	31.30±6.54	14.1±0.46	0.90±0.30	0.60±0.08	5.60±0.98	0.018	0.118
PBS-2	52.00±1.88	31.10±0.86	12.80±1.48	0.80±0.15	0.50±0.28	4.27±0.65	0.015	0.082
PBS-5	54.00±5.71	32.00±7.22	4.90±0.70	0.50±0.53	0.60±1.04	4.80±0.11	0.009	0.089

Table S3 Component of corrosion products on the pure Zn surface (At.%)

Solutions	Immersion time	Zn	C	O	P	N
PBS-0	0.5 h	14.40	29.12	37.76	16.80	1.92
	48 h	8.79	35.96	39.70	13.91	1.63
PBS-0.1	0.5 h	3.73	58.42	23.46	7.76	6.63
	48 h	6.13	42.75	30.16	10.23	10.72
PBS-2	0.5 h	3.92	54.09	24.87	6.82	10.30
	48 h	3.67	50.74	26.47	8.29	10.83

Table S4 The parameters related to Langmuir adsorption isotherm

Solutions	BSA concentration, c, 10^{-8} mol L^{-1}	$i_{corr,c}$ $\mu A\ cm^{-2}$	$c \times 10^{-8} / i_{corr,c}$
PBS-0.05	0.75	0.93	0.81
PBS-0.1	1.50	1.44	1.05
PBS-0.5	7.52	2.86	2.63
PBS-1	15.05	2.84	5.30
PBS-2	30.10	4.43	6.97
PBS-5	75.24	7.23	1.41
